# FDM 3D Printing and Properties of PBS/PLA Blends

**DOI:** 10.3390/polym15214305

**Published:** 2023-11-02

**Authors:** Wangwang Yu, Liwei Sun, Mengya Li, Meihui Li, Wen Lei, Chaohui Wei

**Affiliations:** 1 School of Mechanical Engineering, Nanjing Vocational University of Industry Technology, Nanjing 210023, China; 2 College of Science, Nanjing Forestry University, Nanjing 210037, China

**Keywords:** poly(lactic acid), poly(butylene succinate), blend, fused deposition molding, 3D printing, property

## Abstract

Poly(lactic acid) (PLA) and Poly(butylene succinate) (PBS) were chosen as raw materials and melt blended by a twin screw extruder and pelletized; then, the pellets were extruded into filaments; after that, various PBS/PLA blending samples were prepared by Fused Deposition Molding (FDM) 3D printing technology using the filaments obtained and the effect of the dosage of PBS on technological properties of 3D-printed specimens was investigated. For comparison, the PLA specimen was also prepared by FDM printing. The tensile strength, tensile modulus, thermal stability, and hydrophilicity became poorer with increasing the dosage of PBS, while the flexural strength, flexural modulus, impact strength, and crystallinity increased first and then decreased. The blend containing 10% PBS (10% PBS/PLA) had the greatest flexural strength of 60.12 MPa, tensile modulus of 2360.04 MPa, impact strength of 89.39 kJ/m^2^, and crystallinity of 7.4%, which were increased by 54.65%, 61.04%, 14.78%, and 51.02% compared to those of printed PLA, respectively; this blend also absorbed the least water than any other specimen when immersed in water. Different from the transparent PLA filament, 10% PBS/PLA filament presented a milky white appearance. The printed 10% PBS/PLA specimen had a smooth surface, while the surface of the printed PLA was rough. All the results indicated that the printed 10% PBS/PLA specimen had good comprehensive properties, including improved mechanical properties, crystallization performance, and surface quality than PLA, as well as proper wettability and water absorption. The prominent conclusion achieved in this work was that 10% PBS/PLA should be an ideal candidate for biodegradable feedstock among all the PBS/PLA blends for FDM 3D printing.

## 1. Introduction

3D printing is an emerging manufacturing technology covering a process of manufacturing physical items based on digital 3D model data; this process makes it possible to produce some products with innovative designs and a combination of materials that could not be realized by any other processes [1]. This technique has been used in many areas, including agriculture, civil engineering, packaging, architecture, building, healthcare, biomedical, aviation industry, transportation, and foodstuff [2,3].

As one of the most common 3D printing technologies that can be used to make both daily supplies and industrial products, fused deposition modeling (FDM) has attracted engineers and scientists greatly in recent years because of its wide range, non-toxicity of materials, convenient operation, high production efficiency, little maintenance needed, simple structure of equipment, powerfulness to provide durable printing procedures, simple operation, high strength and toughness of materials [4,5]. It can be performed on a wide range of thermoplastic polymers or composites to allow a filament to be extruded through a small nozzle and build up an object in a melted/softened state on a predefined route layer-by-layer [6]. The polymers or their composites concerned currently include poly(lactic acid) (PLA) [7,8], polybutylene adipate terephthalate [9,10], poly(vinyl chloride) (PVC) [11], thermoplastic polyurethane elastomer [12], polypropylene [13,14], nylon [15,16], polyetheretherketone [17], polycaprolactone (PCL) [18,19], polyhydroxybutyrate [20], polyamide [6,21], acrylonitrile butadiene styrene (ABS) [6,22], and polyphenylene sulfone [4]. PLA is the best choice for FDM 3D printing owing to its low melting point, excellent mechanical properties, thermal stability, biodegradability, non-toxicity, slight shrinkage during processing, good adhesion to the platform, and good dimensional stability of the printed specimens. Besides these, the market for PLA is thought to be one of the two biopolymers expected to grow the most remarkably [6,7,23].

However, PLA by itself has shortcomings; it is relatively hard, its toughness, flexibility, and elasticity are not so good, its printed specimens are easy to be broken, and it also has poor appearance quality. All these limit the widespread application of PLA in the 3D printing area. It is thus of great significance to modify PLA to improve its weak points and meet the requirement for 3D printing; one of the primary methods is by blending PLA with other polymers [24].

As a typical biopolymer, PBS not only has good mechanical properties but also can be processed conveniently [25] and degrades completely. Blending PBS with PLA can increase the ductility of PLA without loss of biodegradability [26,27]. Thus, PBS was often applied to modify PLA when the PLA products were produced by some traditional processes, such as extrusion, injection molding, and compression molding. For example, Sakaowduen et al. [28] developed PBS/PLA blends by extrusion and compression molding and evaluated the mechanical performances of five different PBS/PLA (*w*/*w*) blends and compared them with those of PLA. The impact strength of each blend was greater than that of the PLA when highly flexible PBS was blended with PLA. However, the tensile performances became poorer. Suparanon et al. [26] prepared flame-retardant PBS/PLA blends by a compression molding process and experimented with the effects of flame-retardant agents, dosage of PBS, and combination of the two on the physico-mechanical properties of the blends. They found that the impact toughness of the blends containing 20 wt% PBS rose to about 244% that of the PLA, and an increased content of PBS would lead to a significantly improved elongation at break, impact toughness, and the thermal stability of PLA. Saowaroj Chuayjuljit et al. [29] melt blended PBS and PLA using a 20 mm corotating twin-screw extruder; the extrudates were pelletized and then hot compressed into plates. The impact strength, elongation at break, degree of crystallinity, and thermal stability of PBS/PLA blends were all greater than those of the PLA, and the increment in these properties was dependent on the dosages of PBS, but the tensile strength, tensile modulus, and bending strength all were reduced when more PBS was introduced.

Considering that the PBS/PLA products have much better toughness than that of pure PLA by the traditional processes, in order to solve the drawbacks of neat PLA as the printing material and get the samples with high appearance quality, in this article, the blend was introduced as filaments for FDM 3D printing technology, deep properties, and structural characterization were done on the samples containing various contents of PBS to ensure that the blends could serve as an ideal feedstock for FDM 3D printing.

## 2. Experimental

### 2.1. Materials

PLA pellet(3052D, American Nature Works Co., Minnetonka, MN, USA) was obtained from Suzhou Benfuzhong Plastic Import and Export Co., Ltd., China (Suzhou, China); PBS, TH803S, was obtained from Xinjiang Blue Ridge Tunhe Sci. & Tech. Co., Ltd. (Changji City, Xinjiang, China).

### 2.2. Sample Preparation

PLA and PBS were oven-dried at 60 °C to constant masses to release the adsorbed water. Then, they were melt blended by the proportions as shown in Table 1 using a twin-screw extruder (SHJ-20, Nanjing Giant Machinery Co., Ltd., Nanjing, China) in the temperature range between 165 °C and 180 °C. The rotating speed of the screw was 20 rpm, and the extrudates were pelletized and put into a single screw extruder (KS-HXY, HUANXINYANG Electrical Equipment Co., Ltd., Suzhou, China) to prepare the mixed filament whose diameter was controlled within 1.75 ± 0.05 mm.

The prototypes of the printed specimens were built in CAD software(Auto CAD 2020 Simplified Chinese) and exported as an STL file. The STL file was further sliced into a data file by layers, which was made of Gcode. The STL file was transferred to the printer (MOSHU S108; Hangzhou SHINING 3D Technology Co., Ltd., Hangzhou, China) to make the samples.

We once systematically investigated the effect of printing parameters on the properties of FDM 3D-printed composites and got the optimal printing conditions, as shown in Table 2. Thus, these parameters were adopted in this study to print the samples. Printing was carried out in a standard laboratory without temperature or humidity control.

### 2.3. Testing and Characterization

#### 2.3.1. Mechanical Testing

The mechanical performances were tested in air at room temperature, among which the tensile test was done referring to ASTM D 638-2010 at a cross-head speed of 10 mm/min, and the bending test was done referring to ASTM D 790-2010 at a cross-head speed of 5 mm/min on a universal mechanical testing machine (E44.304, MTS Industrial Systems (China) Co., Ltd., Shenzhen, China) with a 20 kN load capacity. The impact test was done referring to Chinese National Standard GB/T 1043.1-2008 [31] with a pendulum electronic impact testing machine (XJC-25D, Chengde Precision Testing Machine Co., Ltd., Chengde, China).

#### 2.3.2. Morphological Characterization

The morphology of the surface or the fractured surface of the printed item was observed by using a field-emission scanning electron microscope (SEM) (Hitachi SU 8010, Hitachi Corporation, Tokyo, Japan) at an accelerating voltage of 3 kV. For a better resolution, the surface of each sample was coated with gold before the SEM observation.

#### 2.3.3. Thermal Stability Assessment

TGA was realized by employing a thermo-gravimetric analyzer (NETZSCH-Gerätebau GmbH, Selb, Germany) under a nitrogen atmosphere. The printed specimens (6–8 mg) were heated from 20 °C to 550 °C using a heating rate of 20 °C/ min. The onset temperature at which the sample began to decompose, and the peak temperature at which the specimen decomposed the fastest were investigated to assess the effect of the PBS content on the thermal stability of the printed items.

#### 2.3.4. Melt and Crystallization Behavior

The melt and crystallization behavior test was done in three steps on a differential scanning calorimetry (DSC) instrument (DSC214, NETZSCH-Gerätebau GmbH, Selb, Germany). First, the 5–10 mg sample was heated from room temperature to 220 °C at 15 °C/min under a nitrogen atmosphere and held isothermally for 5 min; then, the specimen was cooled down to room temperature. Finally, the sample was reheated to 220 °C. The transition temperatures and heat capacities were obtained using the NETZSCH analysis software (Proteus70). The crystallinity was calculated using the following equation, considering the polymer fraction in the printed sample:

(1) $$x_{c}=\frac{\left|\Delta H_{m}+\Delta H_{cc}\right|}{\omega \Delta H^{*}}$$

where x_c_ represented the degree of crystallinity of the specimen, ω was the mass content of PLA in the specimen, ΔH_m_, ΔH_cc_, and ΔH* were the enthalpies of melting, cold crystallization and melting of 100% crystalline PLA, which was found to be 93 J/g in the literature [2,32,33].

#### 2.3.5. Visual Appearance Observation

For comparison, the filaments and the printed samples of neat PLA and 10% PBS/PLA samples were put on a desk with a black background, and then the filaments and the surfaces of the printed items were photographed using a mobile phone with a resolution of twelve million pixels.

#### 2.3.6. Wettability Testing

The contact angle (θ) of distilled water drops on the surface of each FDM 3D-printed specimen was measured at room temperature using a contact angle instrument (DSA100; KRÜSS GmbH, Borsteler Chaussee, Hamburg, Germany). A 5 µL droplet of distilled water was dropped onto the surface and kept for 15 s, and then the θ values were read at various points.

#### 2.3.7. Water Absorption Testing

Water absorption was tested referring to the ASTM D570-98 [34]. The samples were put into a bath containing tap water at room temperature; after 24 h, the samples were taken out from the water, and the water adsorbed onto the surfaces of the samples was removed using an absorbent lint-free cloth; after that, the samples were weighed in a balance whose precision was 0.1 mg. Next, the samples were submerged in water again. The water absorption x_t_ was obtained using Equation (2), where w_0_ and w_t_ denoted the initial dry mass and the current mass of the samples, respectively. The test was repeated five times, and the averaged values were recorded as the final result.

(2) $$x_{t}=\frac{w_{t}-w_{0}}{w_{0}}\times 100\%$$

#### 2.3.8. Melting Flow Rate Determination

The melting flow rate (MFR) of the specimens was tested referring to Chinese National Standard GB/T 3682-2000 [35] to clarify the flowability of the specimens. The samples were weighed and added to the melt index meter (XNR-400, Chengde Jinhe Instrument Manufacturing Co., Ltd., Chengde, China). The MFR was measured at 160 °C and 2.16 kgf.

## 3. Results and Discussion

### 3.1. Mechanical Performances

**Figure 1:**
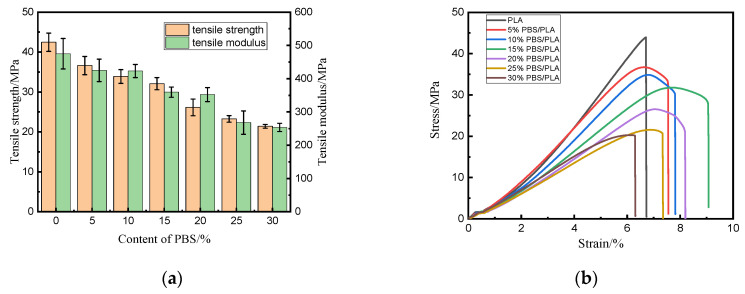

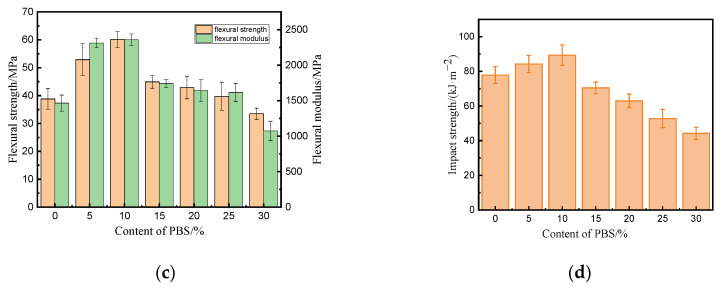
Mechanical performances of PLA and PBS/PLA blends: (**a**) tensile properties; (**b**) σ-ε curves; (**c**) flexural properties; (**d**) impact strength.

The strengths and elongation at the break of the 3D-printed specimens were plotted in Figure 1. It was revealed that the dosage of PBS had a great effect on the tensile, flexural, and impact properties of the 3D-printed PBS/PLA samples.

The PLA has a relatively brittle behavior with a tensile strength of 42.46 MPa, as illustrated in Figure 1a, which was consistent with the value of 42.27 MPa of the previous study [36]. All the 3D-printed PBS/PLA blends exhibited reduced tensile strength compared to PLA, and the tensile strength decreased monotonically when the PBS content increased; a similar changing trend was once concluded by Justyna et al. [37]; this should be caused by the poor interfacial bonding between PLA and PBS, and also by the low stiffness and ductility of PBS. A similar conclusion could be drawn for the tensile modulus. According to stress–strain curves, with the addition of PBS to PLA, the elastic modulus decreased, indicating that the polymer was softened by PBS. When the content of PBS in the blend was 30%, the tensile strength and modulus of the specimens decreased to 21.41 MPa by 49.58% and 253.66 MPa by 46.58% from those of pure PLA, respectively. The stress–strain curves shown in Figure 1b indicated that pure PLA broke in a brittle manner, while the stress (σ) vs. strain (ε) curve of each PBS/PLA blend could be divided into two parts: a quasi-linear section was observed at first which corresponded to the PBS/PLA interface debonding. Then, a reduction of stress combined with an increasing strain revealed a quasi-ductile behavior; a yield point appeared in each curve of the blends. Furthermore, a greater content of PBS made the blend have a lower yield strength. The enhanced toughness might be because the PBS was a flexible filler and a softer material [26]. It was worth mentioning that the fracture mode of PLA had been changed when PBS was introduced, but no obvious alternation happened to the elongation at the break of the specimen.

For the bending and impact properties, the highest values were all found in the 10% PBS/PLA specimen, followed by the 5% PBS/PLA specimen, meaning that the 10% PBS/PLA blend exhibited the best toughening effect compared to neat PLA, which was a pure polymer. The flexural strength, flexural modulus, and the impact strength of the 10% PBS/PLA blend were 60.12 MPa, 2360.04 MPa, and 89.39 kJ/m^2^; a 54.65%, 61.04%, and 14.78% increase from those of neat PLA, respectively, while they were only 33.50 MPa, 1074.35 MPa, and 44.20 kJ/m^2^ for 30% PBS/PLA specimens.

The changing trend of flexural properties was a little different from that of the tensile properties; this might be attributed to the different fracture mechanisms between them. When it was fractured during the flexural test, the sample was actually damaged by compression, shear, and tension simultaneously [38]. Regarding the impact strength, when few PBS was incorporated, it would act as the center of built-in stress and absorb some energy when the sample was affected by an impact force. Thus, the impact strength would increase. When more PBS was introduced, however, the defects arose from the poor interfacial bonding between PBS and PLA, which would reduce the impact strength. As a result, the impact strength first rose and then dropped gradually with the increasing dosage of PBS.

### 3.2. Morphology

**Figure 2:**
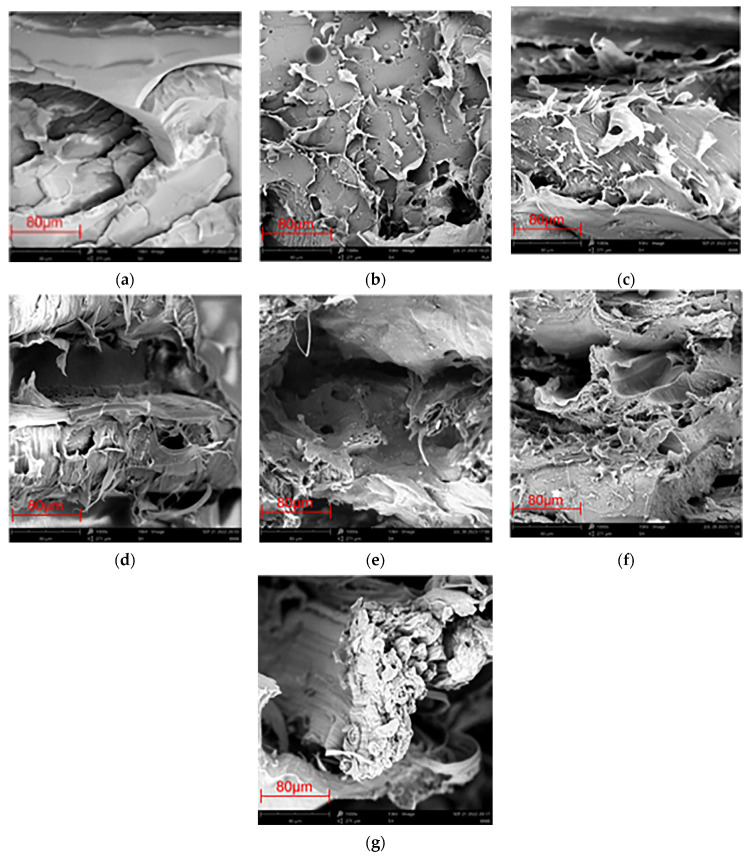
SEM Images of neat PLA and various PBS/PLA blends: (**a**) PLA; (**b**) 5% PBS/PLA; (**c**) 10% PBS/PLA; (**d**) 15% PBS/PLA; (**e**) 20% PBS/PLA; (**f**) 25% PBS/PLA; (**g**) 30% PBS/PLA.

Figure 2 shows the microscopic morphologies of the fracture surfaces of PLA and PBS/PLA blends. The fracture surface of the original PLA was smooth and flat, as observed in the SEM images (Figure 2a), indicating that PLA broke in a brittle manner; this type of fracture manner had previously been reported by some literature [39,40]. After blending with PBS, the microscopic morphologies of the samples containing 5–30% PBS are shown in Figure 2b–g, which are a little rougher than that of neat PLA; in these situations, the blends broke in a ductile manner. This was also in accordance with results from the stress–strain testing, proving that the relative dosage of PBS to PLA affected the mesostructural quality of the obtained products directly. Similar to that of the 3D-printed PCL/PVC blend [41], when PBS content was lower (5% and 10%), the PBS/PLA compounds were single-phase and confirmed that the high molecular affinity of PBS and PLA caused the formation of two miscible compounds. In these two blends, PLA should act as a continuous phase and PBS as the disperse phase, with PBS well dispersed in PLA. However, when more PBS was adopted, partial immiscibility and some defects, such as cleavage and severe plastic deformation, could be observed in the blends, and these defects became more serious when more PBS was applied. The mechanical performances of the specimens were accordingly affected greatly, and the tensile strength of the specimen was thus decreased.

### 3.3. Thermal Stability

**Figure 3:**
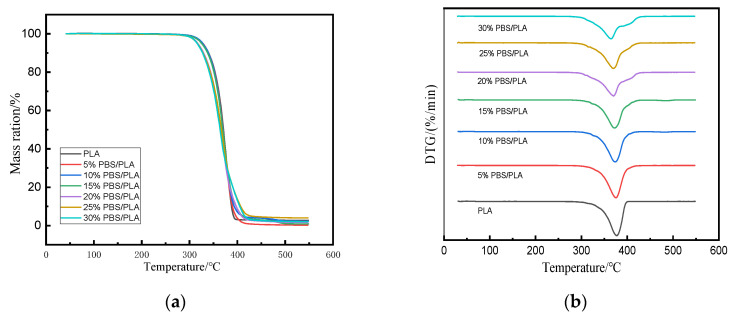
Pyrolysis process of PLA and PBS/PLA blends: (**a**) mass loss curve; (**b**) differential thermogravimetric curve.

Thermal stability was a critical parameter in the performance evaluation of 3D printing materials [42]. The thermal stability of PLA and PBS/PLA blends was investigated using a hermos-gravimetric analyzer. Figure 3 shows the TGA thermograms and the derivative weight loss in TGA thermograms of samples. All the samples decomposed mainly ranging from 250 to 550 °C; the onset temperature (T_i_), the peak temperature at which the sample decomposed fastest (T_p_), and the char residue after decomposition are summarized in Table 3.

PLA began to decompose at 351.8 °C, which was faster between 360–400 °C, and the T_p_ was 377.7 °C. The PBS/PLA blends showed similar trends in thermal degradation with increasing temperature. The thermal degradation of these blends started at lower initial temperatures of thermal degradation and continued to 390–420 °C; this might be due to the lower T_i_ value of PBS (300 °C) [43] than that of PLA. In the DTG curves, PLA, 5%PBS/PLA, 10%PBS/PLA, or 15% PBS/PLA all had one peak between 350–450 °C. However, the 20%PBS/PLA, 25%PBS/PLA, and 30%PBS/PLA all exhibited two peaks; the temperatures corresponding to the main peaks were 370.1 °C, 369.4 °C, and 365.5 °C, respectively; meanwhile, a shoulder peak around 408 °C could be observed on the right of the main peak for these three specimens, the main and shoulder peaks might correspond to the thermal decomposition of PLA and PBS. Furthermore, the shoulder peak became more visible with the increasing dosage of PBS in the blend, indicating that the phase separation between the two polymers became more serious. These results demonstrated that blending with PBS had a negative effect on the thermal stability of PLA, and the samples became more unstable when the PBS content was increased; blending a small amount of PBS with PLA, the two polymers were miscible when more PBS was used. However, the interfacial compatibility and cohesion between the two polymers became poorer.

### 3.4. Melt and Crystallization Behavior

**Figure 4:**
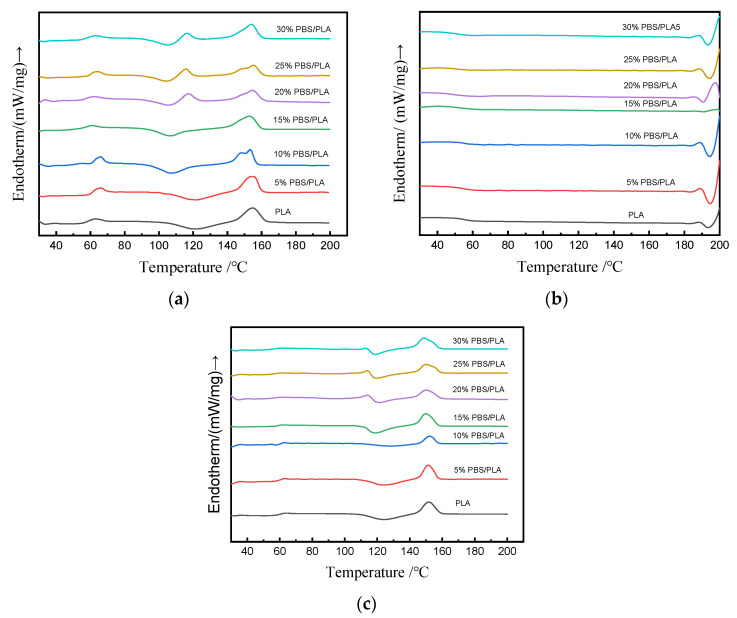
DSC thermograms of printed samples with different contents of PBS: (**a**) first heating, (**b**) cooling, (**c**) second heating.

Because of the slow crystallization rate of PLA, the crystallization performance of PLA has attracted much attention [32]. The heating-run curves of the various PBS/PLA blends obtained by the DSC analysis are shown in Figure 4. The total DSC characteristics of the PBS/PLA blends are summarized in Table 3.

It can be found in Figure 4 that all the samples show no melting-crystallization peak during the cooling stage, and a big cold-crystallization peak appeared during the second heating process. The reason could be that it was hard for PLA to organize its molecular chains in a timely manner, and thus, it would crystallize very slowly [32]. The calculated results in Table 4 reveal that the cold-crystallization temperature first rose, then dropped when the dosage of PBS increased. The T_cc_ for neat PLA was 124.2 °C, while that for the 10%PBS/PLA was 128.2 °C, a 4 °C increase from that of PLA, which was also the greatest among all the blends. The higher T_cc_ meant the 10%PBS/PLA started to crystallize earlier than PLA, indicating that a small amount of PBS could promote the crystallization of PLA [27,44]. When more PBS was adopted, the crystallization of PLA might be hindered by PBS because of the aggregation occurring between PBS molecules. This could also be evidenced by the calculated degree of crystallinity in Table 3; the X_c_ values of the samples rose slightly at first and then reduced with the increase of the mass fraction of PBS in the blend. The 10% PBS/PLA blend had the greatest X_c_ value of 7.4%.

The glass transition temperature (T_g_), shown in Table 3, shows that the T_g_ of neat PLA was 63.8 °C, which was very close to the values of 65.0 °C reported by Marie-Joo et al. [1] and 60.9 °C reported by Yelda et al. [45], and the observation of T_g_ changing for the blends showed that the glass transition temperatures of PLA would be reduced slightly with the increase of PBS content in the blend. For example, T_g_ for the blend [PBS/PLA] = [10/90] was 62.9 °C, and for the blend [PBS/PLA] = [20/80], T_g_ reduced to 61.7 °C, which were 0.9 °C and 2.1 °C lower than that of pure PLA, respectively. The reason was that the content of PBS in the printed samples was relatively low and thus could disperse well in the blend, leading to partial miscibility of the polymers in the amorphous phase [37]; the mobility of the polymer chains was thus less restricted.

### 3.5. Visual Appearance

The print quality of the samples was affected by many factors, and the two most important factors were the printing parameter [46] and the composition of the blends. We once optimized the printing parameters such as printing speed, printing angle, nozzle temperature, platform temperature, and layer thickness. In this research, the samples were prepared on the optimizing printing conditions, and the emphasis was thus put on the effect of the composition of the blends on the print quality.

**Figure 5:**
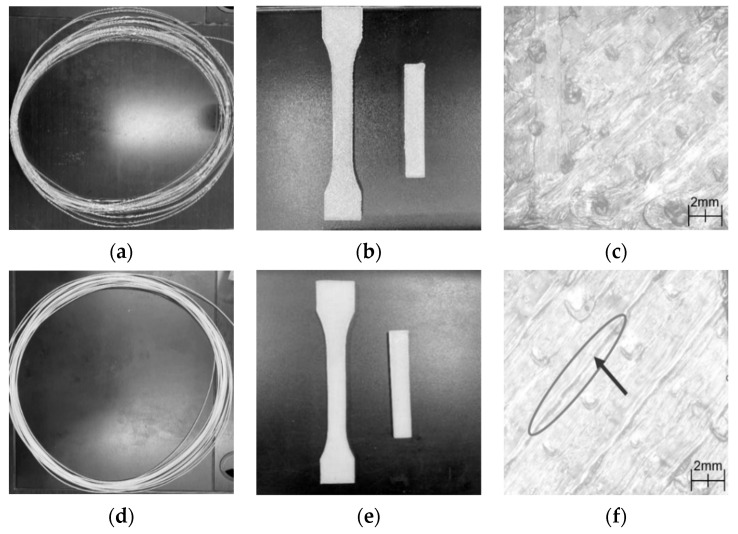
Visual appearance of (**a**) PLA filament; (**b**) printed PLA; (**c**) 10% PBS/PLA filament; (**d**) printed 10% PBS/PLA; and surface morphology: (**e**) printed PLA; (**f**) printed 10% PBS/PLA.

As aforementioned, 10% PBS/PLA had the best interfacial bonding between PBS and PLA among all the printed blends; PBS could be fused into PLA the best. Meanwhile, it had improved flexural and impact properties, as well as increased crystallinity than pure PLA. In this part, the visual appearances of PLA and 10% PBS/PLA were comparatively observed, as illustrated in Figure 5.

Figure 5a,b shows that the PLA filament was transparent, and its printed sample had an uneven surface. However, the 10% PBS/PLA filament was milky white (Figure 5c), and its surface became much smoother (Figure 5d). The SEM observation with a magnification of 40 times indicated that the adjacent filaments in the printed 10% PBS/PLA specimen could bind together tightly (Figure 5f); for the printed PLA specimen, however, many protruding small dots were produced due to the overflow of the PLA melt (Figure 5e). All these indicated that blending the proper amount of PBS with PLA could improve the appearance quality of the printed samples.

### 3.6. Wettability

**Figure 6:**
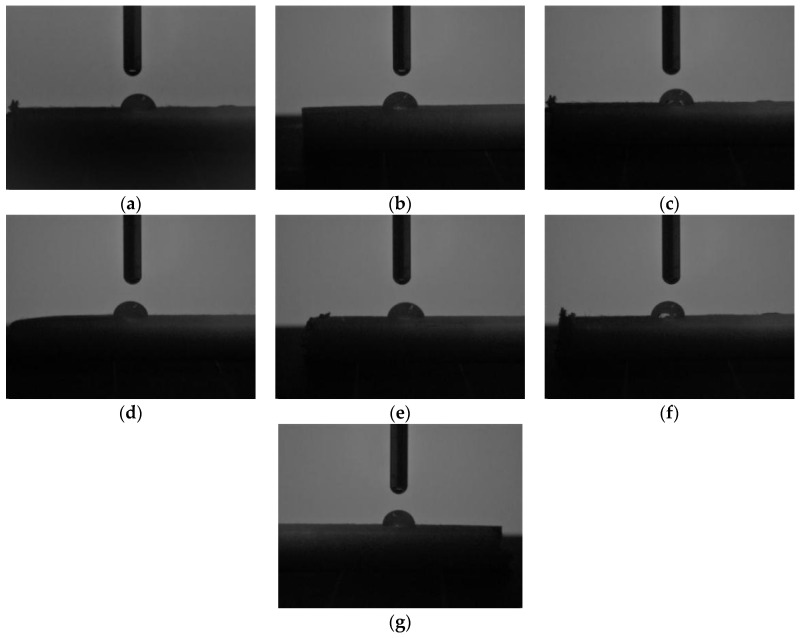
Contact angle images of distilled water on the surfaces of obtained specimens: (**a**) PLA; (**b**) 5% PBS/PLA; (**c**) 10% PBS/PLA; (**d**) 15%PBS/PLA; (**e**) 20% PBS/PLA; (**f**) 25% PBS/PLA; (**g**) 30% PBS/PLA.

The shapes of the water droplets on the surfaces of the specimens are illustrated in Figure 6, and the corresponding contact angles are tabulated in Table 5.

It could be noticed that, on the one hand, the water contact angle on the surface of each sample was smaller than 90 °, indicating that the surfaces of all the samples were hydrophilic and could be wetted with water. On the other hand, the surface wettability of the samples varied with the dosage of PBS; the water contact angles for PLA in Figure 6a and Table 4 were 70.8°. After being blended with PBS, the surface contact angle of the blends increased; in addition, the value showed a monotonic increasing trend. The 30% PBS/PLA had a contact angle of 83.4°, which was 12.6° increased remarkably from that of pure PLA, indicating that the hydrophobicity of the specimens was enhanced. The inducement of PBS improved the hydrophobicity of PLA significantly, which might be due to the introduction of a large amount of hydrophobic ester groups in PBS.

During the production of the blends, PBS was assumed to behave as a solid phase and PLA as a liquid phase; when the surface free energy of the fluid was equal to or lower than that of the solid, the two phases would fuse well [47]. The surface free energy was caused by intermolecular interactions at an interface, and it had a relationship with and could be obtained by measuring contact angles [48]. Thus, the increase in the contact angles of the water droplets on the surfaces of the specimens due to the increasing dosage of PBS could reflect the interfacial compatibilities to some extent; a gradual increase of contact angles meant poorer interfacial compatibility and the mutual fusion between PLA and PBS became more unstable.

### 3.7. Water Absorption

**Figure 7:**
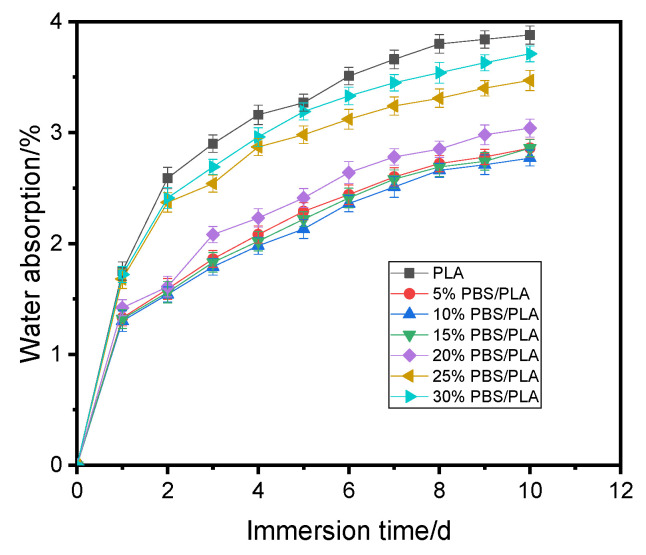
Water absorption curves of different printed samples.

Figure 7 shows the water uptake of the samples immersed in water for 10 days.

Just like those that happened to many composites [49,50], the rate of water absorption by the printed specimens changed with the immersion time. As observed in Figure 7, the samples absorbed water quickly in the first two days and then gradually slowed down; this changing trend was consistent with the Fickian diffusion curve [49]. After immersion in water for 10 days, the water uptake by each sample almost reached equilibrium. The results revealed in Figure 7 demonstrated that all the blends took less moisture than PLA, and at any immersion stage, the water absorption was reduced first with the increase of the PBS content, then rose gradually; 10% PBS/PLA always absorbed the least water. After ten days, the water uptake values of 10% PBS/PLA was only 2.77%, which was lower by 28.61%, 3.25%, 3.25%, 8.88%, 20.17%, and 25.34% than PLA, and 5% PBS/PLA, 15% PBS/PLA, 20% PBS/PLA, 25% PBS/PLA, and 30% PBS/PLA, respectively.

Many factors would affect the water absorption by the printed samples, such as infill density, the composition of the sample, the relative dosage of different components, and the interfacial compatibility [50]. For the printed blend samples in this study, they were all prepared using the same two polymers by the same method. Thus, the relative dosage of PBS to PLA and the interfacial compatibility were critical for the difference in water absorption. As discussed above, when the content of PBS raised from 0 to 10%, PBS could be dispersed in PLA homogeneously, the interfacial compatibility between the two polymers became better, and the defects induced in the printed samples were reduced. Meanwhile, the crystallinity (x_c_) increased, as evidenced in Table 3, and the joint actions by all these factors led to a smaller water uptake by the specimen. When more PBS was used, however, the interfacial compatibility in the blend became poorer, and the number of defects increased. As a result, the water absorption rose.

### 3.8. Flowability

**Figure 8:**
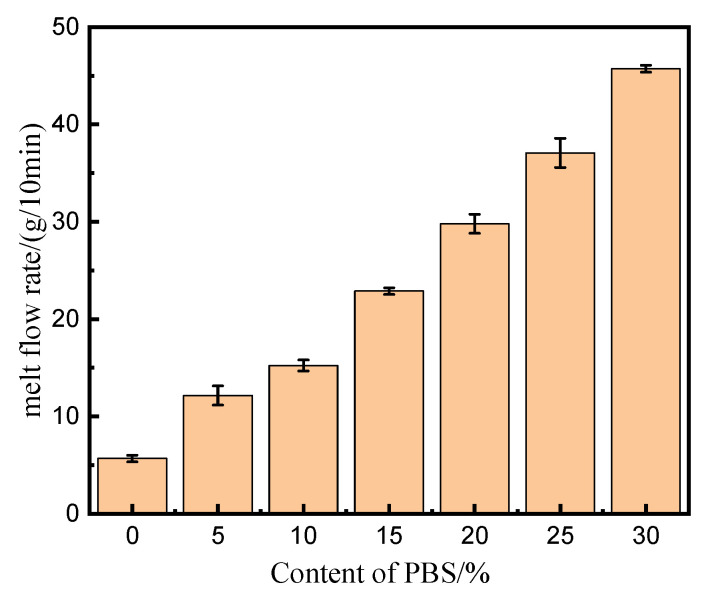
Melt flow rate curves of different samples.

The MFR was investigated to determine the rheological characteristics of the samples. The results are illustrated in Figure 8. It could be found that the MFR value of the samples increased almost linearly with increasing dosage of PBS in the blends; this changing trend had also been observed by Justyna et al. [37] when they investigated the effects of PBS content on the rheological behavior under different temperatures for the injection molding process. The relationship between the dosage of PBS (X) in the blend and the MFR value (Y) of the printed samples could be expressed by the regression equation Y = 4.30 + 1.32X (R = 0.9942). The slope of 1.32 meant that the dosage of PBS affected the flowability of the PLA, obviously. It was well known that the poor flowability of the melt would make the products easily have some defects such as deformation, shark skin, and wrinkles, which would worsen the surface quality of the specimens. When the proper amount of PBS was blended with PLA, the MFR of the polymer was increased, and the flow performance was improved, which was helpful for the smooth printing of the samples. As a result, high-quality products would be obtained; a more uniform and smoother surface could theoretically be observed for the samples. If the value of MFR was too great, however, the melt would flow too fast, leading to a weakened melt strength and, accordingly, poorer mechanical properties. In this study, the 10% PBS/PLA blend had an MFR of 15.22 g/10 min, its melt could flow more smoothly, and the printing performance was much better when compared with neat PLA; thus, the visual appearance of printed samples could be improved, as observed in Figure 5. Meanwhile, its MFR was much lower than those containing more PBS and thus owned more excellent mechanical properties than other samples, as illustrated in Figure 1.

## 4. Conclusions

In this study, PBS was used to modify PLA for FDM 3D printing, and the mechanical properties, fracture surface morphologies, thermal stability, melt and crystallization behavior, wettability, visual appearance, as well as flowability of various samples were investigated. The concluding remarks of this study are as follows:

1. The tensile properties became poorer when more PBS was used. However, the samples containing 10% PBS had the best bending and impact performances; the bending strength, the flexural modulus, and the impact strength of the printed 10% PBS/PLA sample were 60.12 MPa, 2360.04 MPa, and 89.39 kJ/m^2^, which were increased by 54.65%, 61.04%, and 14.78%, respectively, from those of printed PLA samples. After blending with PBS, the fracture of the sample turned from a brittle manner to a little ductile one. SEM observation showed that the fracture surface of the printed PBS/PLA sample became rough while that of the printed PLA sample was smooth. When more PBS was used, serious defects could also be found in the SEM pictures.
2. The printed specimens became more thermally unstable with increasing dosage of PBS. When 10% PBS was used, however, the T_i_ and T_p_ were only reduced by 4.0 °C and 3.7 °C, respectively. When 20% or more PBS was used, a shoulder peak appeared on the right of the main peak in each DTG curve.
3. The samples containing 10% PBS had the greatest degree of crystallinity, the least water absorption among all the samples, and a much better visual appearance than pure PLA.
4. With the increasing dosage of PBS, both the water contact angles and the MFR of the samples increased monotonically.

To sum up, blending with the proper amount of PBS contributes to improving the comprehensive printing performance and the quality of the printed samples, among which the best percentage of PBS should be 10%. The excellent comprehensive performances made the 3D-printed PBS/PLA blends have good market prospects in many areas, such as wearable smart devices, bioengineering, and medical devices.

## Figures and Tables

**Table 1:** Compositions of different blends.

Sample Codes	PLA	5% PBS/PLA	10% PBS/PLA	15% PBS/PLA	20% PBS/PLA	25% PBS/PLA	30% PBS/PLA
PLA/wt%	100	95	90	85	80	75	70
PBS/wt%	0	5	10	15	20	25	30

**Table 2:** Printing parameters of different blends [30].

Parameter	Nozzle Temperature/°C	Platform Temperature/°C	Printing Speed/ mm/s	Layer Thickness/ mm
value	220	50	50	0.1

**Table 3:** Thermogravimetric analysis data of PLA and 3D-printed PBS/PLA blends.

Sample Code	T_i_/°C	T_p,1_/°C	T_p,2_/°C	W/% (550 °C)
PLA	351.8	377.7		2.74
5% PBS/PLA	349.5	375.8		0.25
10% PBS/PLA	347.8	373.9		2.30
15% PBS/PLA	346.0	372.3		0.87
20% PBS/PLA	339.3	370.1	408.33	3.98
25% PBS/PLA	338.5	369.4	407.87	2.27
30% PBS/PLA	333.3	365.5	407.14	2.04

**Table 4:** DSC thermal information of different specimens.

Mass Fraction of PBS/wt%	Tg/°C	Tcc/°C	Tm/°C	ΔHcc/(J/g)	ΔHm/(J/g)	Χc/%
0	63.8	124.2	151.7	−18.53	23.17	4.9
5	63.1	124.6	151.4	−18.96	23.74	5.4
10	62.9	128.2	152.3	−5.83	12.3	7.4
15	61.8	121.9	151.7	−11.71	17	6.7
20	61.7	119.8	150.4	−14.59	18.87	5.7
25	61.3	119.8	150.1	−15.8	19.51	5.3
30	61.2	118.7	148.8	−18.77	21.42	4.1

**Table 5:** Contact angles of distilled water on the surfaces of printed samples with different contents of PBS.

Sample	PLA	5% PBS/ PLA	10% PBS/ PLA	15% PBS/PLA	20% PBS/PLA	25% PBS/PLA	30% PBS/ PLA
Contact angle/°	70.8 ± 0.5	74.1 ± 0.4	75.6 ± 0.6	77.2 ± 0.5	78.1 ± 0.4	79.3 ± 0.4	83.4 ± 0.5

## Data Availability

The data presented in this study are available on request from the corresponding author.
